# Connectivity Map Analysis Indicates PI3K/Akt/mTOR Inhibitors as Potential Anti-Hypoxia Drugs in Neuroblastoma

**DOI:** 10.3390/cancers13112809

**Published:** 2021-06-04

**Authors:** Paolo Uva, Maria Carla Bosco, Alessandra Eva, Massimo Conte, Alberto Garaventa, Loredana Amoroso, Davide Cangelosi

**Affiliations:** 1 Clinical Bioinformatics Unit, Scientific Direction, IRCCS Istituto Giannina Gaslini, Via Gerolamo Gaslini 5, 16147 Genova, Italy; paolouva@gaslini.org; 2 Italian Institute of Technology, Via Morego 30, 16163 Genova, Italy; 3 Laboratory of Molecular Biology, IRCCS Istituto Giannina Gaslini, Via Gerolamo Gaslini 5, 16147 Genova, Italy; mariacarlabosco@gaslini.org (M.C.B.); alessandraeva@gaslini.org (A.E.); 4 UOC Oncologia, IRCCS Istituto Giannina Gaslini, Via Gerolamo Gaslini 5, 16147 Genova, Italy; massimoconte@gaslini.org (M.C.); albertogaraventa@gaslini.org (A.G.); loredanaamoroso@gaslini.org (L.A.)

**Keywords:** neuroblastoma, hypoxia, PI3K/Akt/mTOR inhibitors, treatment

## Abstract

**Simple Summary:**

A large percentage of patients with neuroblastoma relapse and die, despite treatment, thus demanding new personalized strategies and therapeutic targets. Hypoxia, a condition of reduced oxygenation in several solid tumors, has profound effects on the neuroblastoma (NB) tumor biology and patient prognosis. Establishing new connections between hypoxia and pharmacological compounds may provide novel treatment strategies for NB patients. In the present study, we successfully identified 19 compounds mainly belonging to the class of PI3K/Akt/mTOR inhibitors, whose anti-hypoxia effect was shown on the gene expression profile of nine distinct cell lines using connectivity map software. We independently confirmed these findings on NB cells cultured under hypoxia conditions and treated with the mTORC inhibitor PP242. PI3K/Akt/mTOR inhibitors represent a potential effective class of compounds targeting hypoxia in neuroblastoma. PI3K/Akt/mTOR inhibitors may thus find future applicability as a new adjuvant therapy in randomized clinical trials involving neuroblastoma patients with hypoxic tumors.

**Abstract:**

Neuroblastoma (NB) is one of the deadliest pediatric cancers, accounting for 15% of deaths in childhood. Hypoxia is a condition of low oxygen tension occurring in solid tumors and has an unfavorable prognostic factor for NB. In the present study, we aimed to identify novel promising drugs for NB treatment. Connectivity Map (CMap), an online resource for drug repurposing, was used to identify connections between hypoxia-modulated genes in NB tumors and compounds. Two sets of 34 and 21 genes up- and down-regulated between hypoxic and normoxic primary NB tumors, respectively, were analyzed with CMap. The analysis reported a significant negative connectivity score across nine cell lines for 19 compounds mainly belonging to the class of PI3K/Akt/mTOR inhibitors. The gene expression profiles of NB cells cultured under hypoxic conditions and treated with the mTORC complex inhibitor PP242, referred to as the Mohlin dataset, was used to validate the CMap findings. A heat map representation of hypoxia-modulated genes in the Mohlin dataset and the gene set enrichment analysis (GSEA) showed an opposite regulation of these genes in the set of NB cells treated with the mTORC inhibitor PP242. In conclusion, our analysis identified inhibitors of the PI3K/Akt/mTOR signaling pathway as novel candidate compounds to treat NB patients with hypoxic tumors and a poor prognosis.

## 1. Introduction

Neuroblastoma (NB) is one of the deadliest pediatric cancers, accounting for 15% of deaths in childhood [1]. Patients receiving an NB diagnosis are classified into very-low-, low-, intermediate-, or high-risk, according to a pre-treatment risk assignment schema [1]. The risk group determines treatment protocol. The treatment of NB patients ranges from wait-and-see to multimodal therapies involving different combinations of chemotherapy drugs, surgery, autologous transplant, radiotherapy, and disialoganglioside (GD2)-targeted immunotherapy [1]. Even though therapeutic advances of the last years have increased our ability to treat patients affected by NB, 50% of patients who achieve remission later relapse [2] and die, thus demanding new personalized approaches and therapeutic targets [3].

Pathologic hypoxia is a condition of low oxygen tension that occurs in most human solid tumors, including NB, promoting proliferation, angiogenesis, epithelial-to-mesenchymal transition, invasion, metastasis, stem cell maintenance, and resistance to radio- and chemo-therapy [4]. Hypoxia is an unfavorable condition affecting the tumor microenvironment and is a well-known therapeutic target [4,5]. Responses to hypoxia are orchestrated in part through the activation of the hypoxia-inducible-factor (HIF)-1 and HIF-2, which transactivate several hypoxia-responsive genes [6,7]. Hypoxia is detectable in ~25% of NB primary tumors, and is an independent unfavorable prognostic factor for NB, able to significantly stratify clinically relevant subsets of patients, including patients older than 18 months with stage 4 tumors, or with stage 3 and not amplified MYCN tumors [8]. In addition, hypoxia is associated with telomerase activation, a master regulator of telomere maintenance, and a hypoxic, immunosuppressive, poorly differentiated and apoptosis-resistant tumor microenvironment [8]. Hypoxia can be ascertained from the gene expression profile of the tumor, as shown by our and other groups [8,9,10,11,12,13]. Establishing new connections between hypoxia-modulated genes in NB tumors and small molecule compounds may provide novel personalized treatments for an independent subset of NB patients with an unfavorable prognosis.

Connectivity Map (CMap) is an online resource designed to analyze gene pathways, uncover structure–function relationships, and investigate drug repurposing [14,15]. To achieve these goals, the CMap team created a reference database of over 1 million expression profiles of breast, prostate, lung, melanoma, liver carcinoma, and colon cancer cell lines, as well as one immortalized normal kidney epithelial cell line. Profiles have been obtained by stimulating each type of cell line with one of 19,811 small molecule compounds or with one of 5075 genetic perturbations (pertubagens) [16]. The basic assumption implemented by CMap is that if a set of genes, resulting in being differentially expressed between two conditions, is similarly differentially expressed by perturbing a cell line with a given perturbagen, the effect of the perturbagen and that of the disorder of interest may be similar, and denotes a connection between the two signatures [16]. The authors proposed a numeric score for measuring the degree of connectivity between a disorder and a perturbagen [16]. A negative connectivity score reflects the potential effect of that small molecule to reverse the signature of a specific disorder and can, thus, be used as an indicator for therapy [16]. For these reasons, drug repurposing remains one of the most straightforward applications of CMap in biomedical science [16].

In the present study, we aimed to identify novel promising compounds for anti-hypoxia NB treatment using the CMap tool. Our findings identified inhibitors of the phosphatidylinositol 3-kinase/AKT/mammalian target of rapamycin (PI3K/Akt/mTOR) signaling pathway, one of the most aberrantly regulated cellular pathways in cancer [17,18], as novel candidate drugs to treat NB patients with hypoxic tumors.

## 2. Materials and Methods

### 2.1. CMap Analysis

CMap is an online resource potentially capable of discovering new drugs and stimulating new hypotheses of connection among diseases, genes, and therapeutic drugs [16]. The connection between a query signature and a perturbagen in a given cell line is measured by a connectivity score (tau), ranging between +100 and −100, that quantifies the fraction of signatures in the reference database with a greater similarity to the perturbagen than the current query signature. Median_tau_score is a numeric value summarizing the connectivity score between a query signature and a perturbagen across cell lines. Two signatures that have a high positive connectivity score are said to be positively connected and two with a high negative score are said to be negatively connected. Tau scores greater than 90 or lower than −90 between two signatures are considered to have a high connectivity. Query signatures are annotated using the following three categories: (i) the not valid Human Genome Organisation (HUGO) symbol, which is not used in Query (invalid gene); (ii) valid HUGO symbol and part of Best INFerred Genes (BING) space, which is used in Query (valid gene), and (iii) valid HUGO symbol not part of BING space, which is not used in Query (valid but not used in the query) [14]. The gene expression profile in the CMap reference database has been measured by a L1000 high-throughput gene expression assay, which measures the expression of 978 genes referred to as landmark genes in cells treated with perturbagens. The gene expression of a set of 10,174 genes, referred to as BING, which include the 978 landmark genes and 9196 non-landmark genes, are inferred using a computational approach. The CMap database is accessible through a browser at the [19] website or directly via an application programming interface. Public tools may be freely used to interrogate the CMap reference database. Among them, Query is a tool to find connections between a signature in the reference database and query signatures associated with a disease or pathological condition of interest. Connections with specific subsets of perturbagens may be selected using Quick Tools. The Query tool was used to find new connections between hypoxia-modulated genes and therapeutic drugs.

### 2.2. Gene Set Enrichment Analysis

Gene set enrichment analysis (GSEA) [20] was used to assess the enrichment of the gene sets belonging to the custom collection or Hallmark (H) collection retrieved from the Molecular Signature Database (MSigDB) v7.2 database [21]. GSEA calculates an enrichment score (ES) and normalized enrichment score (NES) for each gene set and estimates the statistical significance of the NES by an empirical permutation test using 1000 gene permutations to obtain the nominal *p*-value (NOM *p*-val). When multiple gene sets are evaluated, GSEA adjusts the estimate of the significance level to account for multiple hypothesis testing. To this end, GSEA computes the false discovery rate q-value (FDR q-value), measuring the estimated probability that the normalized enrichment score represents a false positive finding. We considered gene sets containing between 10 and 500 genes. Gene sets with nominal *p*-values lower than 0.05 and FDR q-values lower than 0.05 are considered significantly enriched.

GSEA analysis was performed using the gene expression profiles of SK-N-BE(2)c NB cells cultured under normoxia or hypoxia for 24 h and treated with the mTORC complex inhibitor PP242 or DMSO, as available in the literature [22]. Normalized data were retrieved from the R2: Genomics Analysis and Visualization Platform [23] and were used as the expression data to perform the GSEA analysis.

## 3. Results

### 3.1. The CMap Analysis Identified Significant Connections Between Hypoxia-Modulated Genes and Compounds Belonging to the Class of PI3K/Akt/mTOR Onhibitors

**Figure 1:**
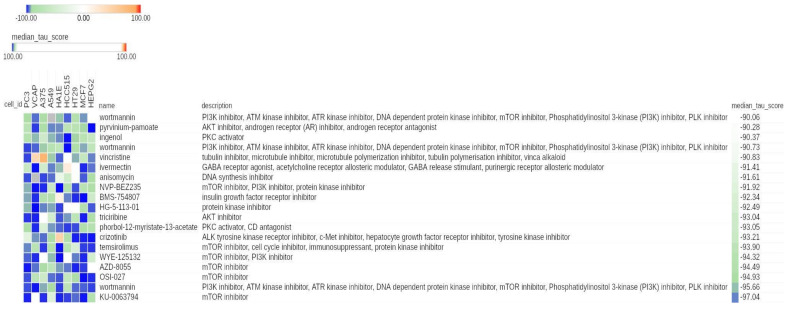
Connectivity Map (CMap) results displaying significant connections between compounds and hypoxia-modulated genes in neuroblastoma (NB). Heat map showing the connectivity score between each cell line (columns) and the most significant compounds (rows). The heat map was generated using the CMap tool. Numeric identifier, compound name, description, and median tau score for each compound are indicated. The cell line identifier is shown on top. The median tau score measures the strength of connection between a query signature and a drug across multiple cell types. Compounds are considered significantly connected with the reference signature when the mean tau score is lower than −90. Compounds are sorted by decreasing order of median tau score. Color keys are displayed on top of the figure.

In a recently published study, we proposed a seven gene hypoxia signature (NB-hop) and identified 34 up- and 21 down-regulated genes between hypoxic/unfavorable (UF NB-hop) and normoxic/favorable (F NB-hop) primary NB tumors [8]. To investigate new connections between hypoxia-modulated genes in NB tumors and novel small molecule compounds, we analyzed the two lists of differentially expressed genes using the CMap tool. CMap annotates Query signatures using the categories valid, valid but not used in Query, and invalid based on HUGO gene nomenclature and best inferred genes in the CMap L1000 platform [16]. Here, 33 up- and 19 down-regulated genes were recognized as valid genes and were used for the subsequent analysis (Table S1). We excluded from the analysis LIN28B, DNER, and SRCIN1, because they were not recognized as part of the BING space (Table S1). The CMap analysis reported a connectivity between hypoxia-modulated genes and 2837 compounds across nine cell lines. The median tau score, the metric summarizing the connectivity among signatures across cell lines, ranged from +78.42 to −97.04 (Table S2). To focus on the compounds with the highest connectivity, we retained those with a median tau score >90 or ≤−90. No compounds displayed a significant positive connectivity, whereas 19/2837 displayed a negative connectivity (Figure 1 and Table 1). Three identifiers were reported for the wortmannin compounds. Therefore, 17 distinct compounds were considered.

Among them, seven included mTOR inhibitor among their mechanisms of action, three included PI3K inhibitors, two included AKT inhibitors, two included PKC activators, one was a tubulin inhibitor (namely vincristine), one was a GABA receptor antagonist, one was a DNA synthesis inhibitor, and one was an ALK tyrosine kinase receptor inhibitor (namely crizotinib; Figure 1). Out of the 17 compounds, 10 displayed more than 1 mechanism of action, indicating a broader spectrum of action (Figure 1). These data evidence a clear negative connection between hypoxia-modulated genes and compounds belonging to the class of PI3K/Akt/mTOR inhibitors, in particular, mTOR inhibitors. For each compound listed in Figure 1, we collected data on the clinical phase, Food and Drug Administration (FDA) approval, and ongoing clinical trials involving NB in Table 1. Four compounds were in a preclinical phase, two were in phase 1, two were in phase 2, one was in phase 3, one was in phase 1/2, and one was undetermined. Whereas, temsirolimus, crizotinib, ivermectin, vincristine, ingenol, and pyrvinium-pamoate are already FDA-approved drugs. Among the latter group, temsirolimus, crizotinib, and vincristine have already been used in some clinical trials involving NB patients [24].

These results prompted us to investigate the connectivity between hypoxia-modulated genes and the entire set of 28 mTOR, 30 PI3K, and 14 AKT inhibitors in the CMap reference database.

**Figure 2:**
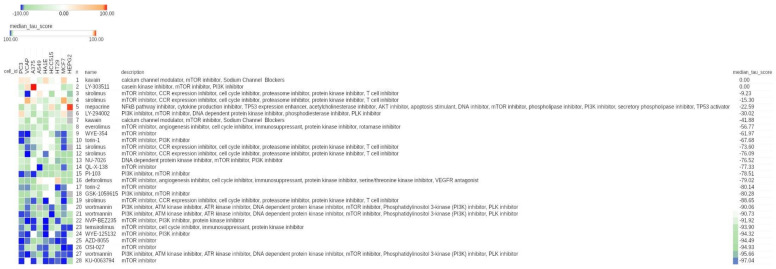
CMap results related to mTOR inhibitors in the entire CMap reference database. Heat map showing the connectivity score between each cell line (columns) and compound (rows). The heat map was generated using the CMap tool. The compounds are relative to the entire class of mTOR inhibitors. The unique numeric identifier, compound name, description, and median tau score of each compound are indicated. The cell line identifier is shown on the top of the map. The median tau score measures the strength of connection between a query signature and a drug across multiple cell types. Compounds are sorted by decreasing order of median tau score. Color keys are displayed on top of the figure.

As shown in Figure 2, we found a certain degree of connectivity for all of the mTOR inhibitors in the CMap reference database.

Although heterogeneous connectivity was detectable across different mTOR inhibitors, 26/28 showed a negative connectivity and only 2/28 displayed a zero value, thereby confirming the negative connectivity between the class of mTOR inhibitors and hypoxia-modulated genes, suggesting that mTOR inhibitors are suitable anti-hypoxia candidate drugs.

Rapamycin/sirolimus is a well-known mTOR inhibitor with reported therapeutic efficacy on aggressive MYCN amplified neuroblastomas [25]. Searching for rapamycin/sirolimus in the CMap results in Figure 2, we found five sirolimus compounds. The most negatively connected sirolimus compound with hypoxia-modulated genes in NB displayed a score of −88, suggesting a high negative connectivity, but lower than other mTOR inhibitors reported in Figure 1 and Figure 2, such as temsirolimus.

**Figure 3:**
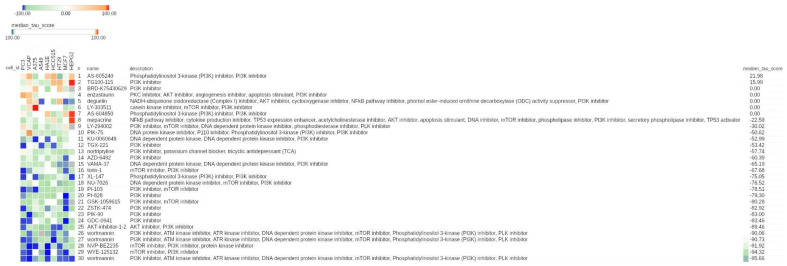
CMap results related to PI3K inhibitors in the entire CMap reference database. Heat map showing the connectivity score between each cell line (columns) and compound (rows). The heat map was generated using the CMap tool. Compounds are relative to the class of PI3K inhibitors. The numeric identifier, compound name, description, and median tau score for each compound are indicated. The cell line identifier is shown on top of the figure. The median tau score measures the strength of connection between a query signature and a drug across multiple cell types. Compounds are sorted by decreasing order of median tau score. Color keys are displayed on top of the figure.

By analyzing the class of PI3K inhibitors, we found that 23/30 compounds displayed a negative connectivity, 5 displayed a 0 value, and 2 displayed a positive value, suggesting a mainly negative connectivity between PI3K inhibitors and hypoxia-modulated genes (Figure 3).

**Figure 4:**
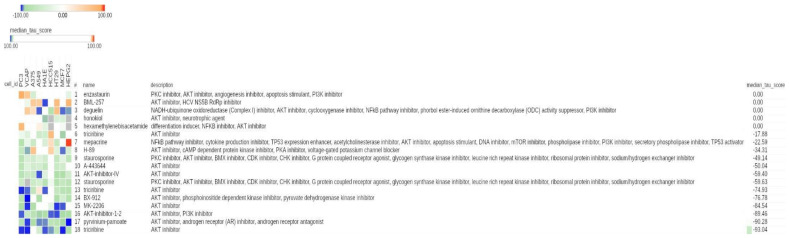
CMap results related to AKT inhibitors in the entire CMap reference database. Heat map showing the connectivity score between each cell line (columns) and compound (rows). The heat map was generated using the CMap tool. Compounds are relative to the class of AKT inhibitors. The numeric identifier, compound name, description and median tau score for each compound are indicated. The cell line identifier is shown on top of the figure. Median tau score measures the strength of connection between a query signature and a drug across multiple cell types. Compounds are sorted by decreasing order of median tau score. Color keys are displayed on top of the figure.

For the class of AKT inhibitors, the results showed that 13 displayed a negative median tau value and 5 displayed a value of 0, confirming the prevalent negative connectivity between AKT inhibitors and hypoxia-modulated genes (Figure 4).

Taken together, our findings evidence that PI3K/Akt/mTOR inhibitors, in particular, mTOR inhibitors, are potentially able to revert the expression of the genes associated with the response to hypoxia in NB tumors.

### 3.2. Validation of CMap Findings in Published Gene Expression Profiles of NB Cells Cultured under Hypoxia

**Figure 5:**
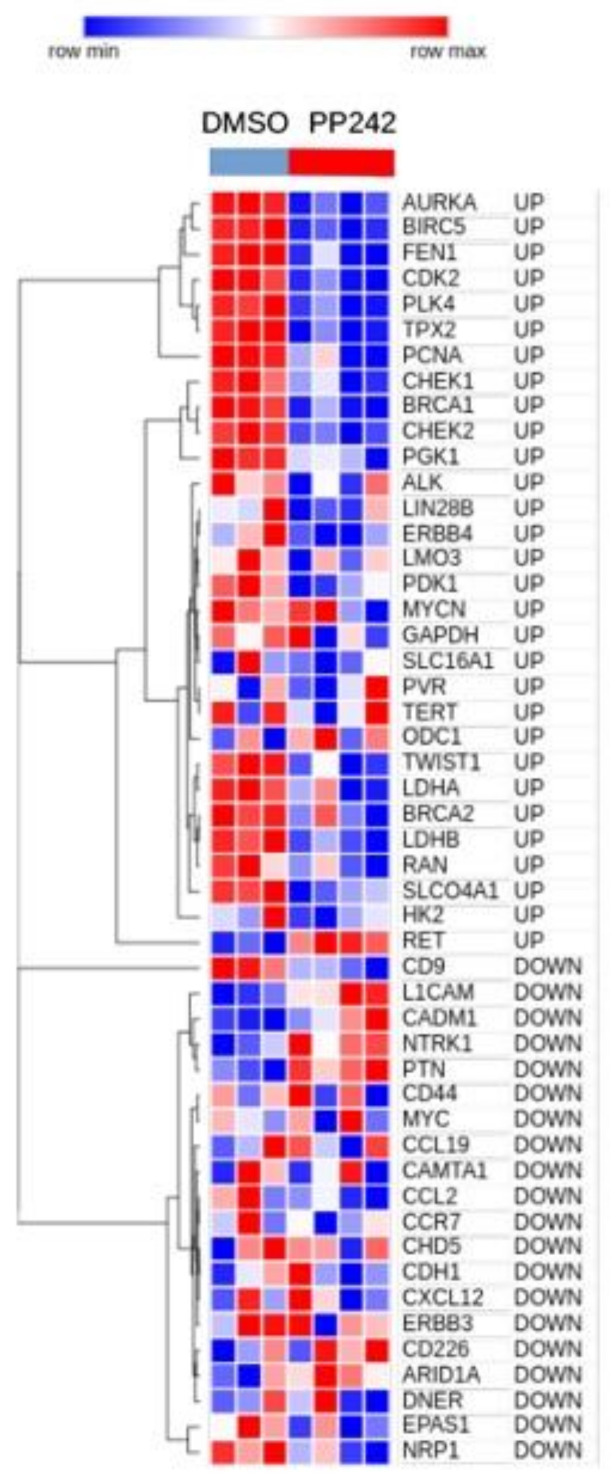
Heat map visualization of hypoxia-modulated genes in the gene expression profile of SK-N-BE(2)c NB cell lines treated with PP242 or dimethyl sulfoxide (DMSO). The heat map was generated using the Morpheus tool. The expression values for each gene modulated in hypoxic NB tumors (rows) are scaled and represented by pseudo-colors in the heat map. Red corresponds to high levels of expression and blue corresponds to low levels of expression. Experimental replicates (columns) are divided into two groups according to treatment. The two compounds used are indicated on top of the heat map. The expression of genes is grouped by hierarchical clustering. The dendrogram is shown on the left. Gene names and type of regulation in hypoxic NB tumors are shown on the right.

Because CMap does not include NB cell lines among those used to create the reference database, we needed to confirm the CMap findings in NB cells. To this aim, we performed a literature search to find the published gene expression profiles of NB cell lines cultured under hypoxia and treated with mTOR inhibitors. Mohlin and colleagues recently published a dataset, hereafter referred to as the Mohlin dataset, which was used to show the therapeutic efficacy of targeting PI3K and mTOR complex 2 (mTORC2) in aggressive NB cells [22]. In this study, SK-N-BE(2)c NB cells treated with the mTORC complex inhibitor, PP242, or dimethyl sulfoxide (DMSO), as a control, were cultured under hypoxic conditions for different time points, and the gene expression profile was determined by microarray analysis [22]. We extracted the gene expression profiles relative to the 24 h of hypoxia treatment from the Mohlin dataset, and used these data to assess the expression of the 34 up- and 21 down-regulated genes analyzed with CMap. CDK1, PKM, PROM1, SRCIN1, and TP53, which we found to be modulated by hypoxia in primary NB tumors [8], were absent in the Mohlin dataset and were, thus, excluded from subsequent analyses (Table S3). A heatmap of those genes found present in the Mohlin dataset revealed that all genes that we had identified as being up-regulated in hypoxic NB tumors [8] are down-regulated after PP242 treatment in the SK-N-BE(2)c NB cell line cultured under hypoxic conditions for 24 h. The inverse behavior was observed for downregulated genes, thereby corroborating the negative connectivity between mTOR inhibitors and hypoxia in NB cells (Figure 5).

**Figure 6:**
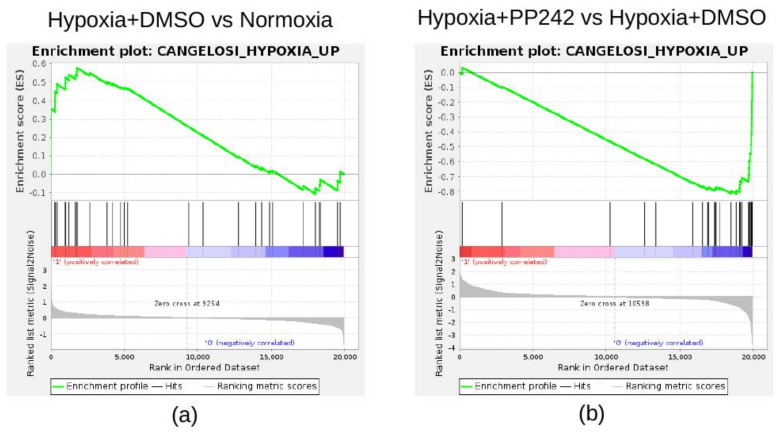
Enrichment plots for custom gene sets found significantly enriched in two distinct gene set enrichment analysis (GSEA) experiments. The plots report the significant enrichment of the CANGELOSI_HYPOXIA_UP gene set in SK-N-BE(2)c cells cultured under hypoxia (**a**) or with mTORC complex inhibitor PP242 (**b**). The experimental groups used to perform GSEA are reported on top of the plots. The enrichment plot shows the gene set name (top), the running enrichment score (green curve), the positions of the gene set hits on the rank ordered list in GSEA (black bars), and the rank ordered list according to the signal-to-noise metric (bottom). Red indicates the highest signal-to-noise values, whereas blue indicates the lowest ones.

We then investigated whether the mTOR inhibitor, PP242, was able to revert the gene expression changes induced by hypoxia in NB cells. To this aim, we carried out a GSEA, which is a powerful method to find the coordinated modulation of functionally related gene sets [20]. Custom collection with two gene sets was created using the 34 up- (CANGELOSI_HYPOXIA_UP) and 21 down-regulated genes (CANGELOSI_HYPOXIA_DOWN) that we reported to be modulated in hypoxic tumors [8]. The gene expression profile of SK-N-BE(2)c NB cells cultured under normoxia and hypoxia treated with DMSO were used to perform the analysis. GSEA evidenced a significant positive enrichment of CANGELOSI_HYPOXIA_UP in the NB cells cultured under hypoxia (*p* < 0.05 and q-values < 0.05; Figure 6A).

The gene expression profile of SK-N-BE(2)c NB cells cultured under hypoxia and treated with mTOR inhibitor PP242 or DMSO were also analyzed using GSEA on the same custom collection for comparison. In the subset of SK-N-BE(2)c cells treated with PP242, GSEA identified a significant negative enrichment of CANGELOSI_HYPOXIA_UP (*p* < 0.05 and q-values < 0.05; Figure 6B).

No significant enrichment was found for CANGELOSI_HYPOXIA_DOWN in the two above analyses. Taken together, these findings provide a clear indication that the mTOR inhibitor, PP242, can revert the expression of the genes induced in hypoxic NB tumors.

Eight collections of gene sets are provided within the MSigDB resources [21]. Hallmark is a curated collection of gene sets suitable for explorative analyses [8,21]. To investigate the biological processes and pathways modulated by hypoxia in the Mohlin dataset, we ran GSEA using the gene expression profile of SK-N-BE(2)c NB cells cultured under normoxia or hypoxia and the Hallmark gene set collection. Nine gene sets were positively enriched in NB cells cultured under hypoxia, whereas two were negatively enriched in the same group (NOM *p*-value < 0.05 and FDR q-value < 0.05; Table 2).

**Figure 7:**
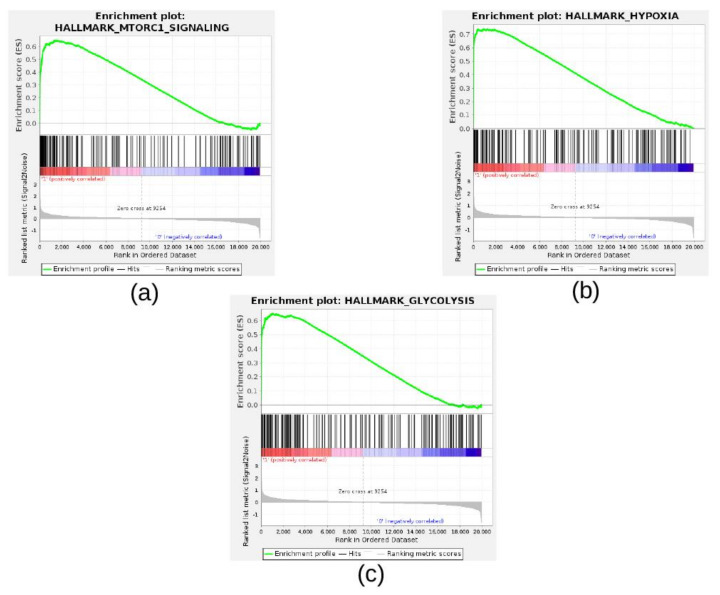
Enrichment plots for selected hallmark gene sets found positively enriched in SK-N-BE(2)c cells cultured under hypoxia and treated with DMSO with respect to SK-N-BE(2)c cells cultured under normoxic conditions. The enrichment plot shows the gene set name (top), the running enrichment score (green curve), the positions of the gene set hits on the rank ordered list in GSEA (black bars), and the rank ordered list according to the signal-to-noise metric (bottom). Red indicates the highest signal-to-noise values, whereas blue indicates the lowest ones. Plots are relative to (**a**) HALLMARK_MTORC1_SIGNALING, (**b**) HALLMARK_HYPOXIA, and (**c**) HALLMARK_GLYCOLYSIS.

Interestingly, among the significantly enriched gene sets of GSEA, we found HALLMARK_MTORC1_SIGNALING, HALLMARK_HYPOXIA, and HALLMARK_GLYCOLYSIS (Table 2). The GSEA enrichment plots of these gene sets highlighted a clear up-regulation of genes involved in the cellular response to hypoxia and MTORC1 signaling in NB cells cultured under hypoxia (Figure 7A–C).

To investigate the biological processes and pathways modulated by the mTOR inhibitor, PP242, in NB cells, we ran GSEA using the gene expression profile of SK-N-BE(2)c NB cells treated with PP242 or DMSO, and the Hallmark gene set collection. Fourteen gene sets were negatively enriched in NB cells treated with PP242 (NOM *p*-value < 0.05 and FDR q-value < 0.05; Table 3), whereas none were positively enriched in the PP242-treated cells.

**Figure 8:**
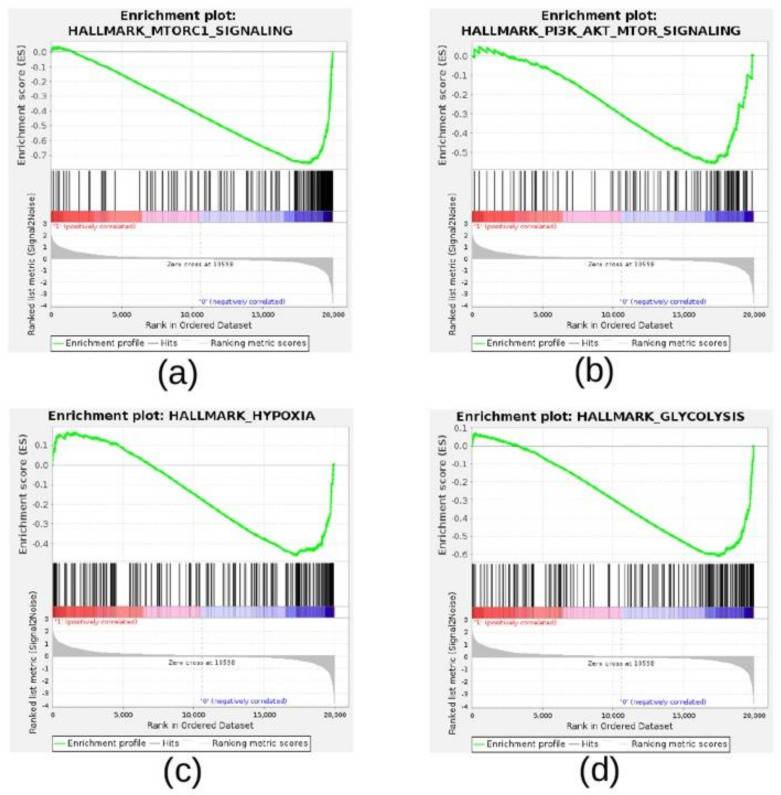
Enrichment plots for selected hallmark gene sets found negatively enriched in NB cells treated with mTORC complex inhibitor PP242. Selected enrichment plots from GSEA. The enrichment plot shows the gene set name (top), the running enrichment score (green curve), the positions of the gene set hits on the rank ordered list in GSEA (black bars), the rank ordered list according to the signal-to-noise metric (bottom). Red indicates the highest signal-to-noise values, whereas blue indicates the lowest ones. Plots are relative to: (**a**) HALLMARK_MTORC1_SIGNALING, (**b**) HALLMARK_PI3K_AKT_MTOR_SIGNALING, (**c**) HALLMARK_HYPOXIA, and (**d**) HALLMARK_GLYCOLYSIS.

Interestingly, we found HALLMARK_MTORC1_SIGNALING, HALLMARK_PI3K_AKT_MTOR_SIGNALING, HALLMARK_HYPOXIA, and HALLMARK_GLYCOLYSIS among the negatively enriched gene sets (Table 3). The GSEA enrichment plots of these gene sets highlighted a clear inhibition of the PI3K/AKT/MTOR pathway and the cellular response to hypoxia across NB cells treated with PP242 (Figure 8A–D).

Furthermore, in the same group of cells treated with PP242, we observed the inhibition of gene sets involved in cell cycle, DNA repair, metabolism, and apoptosis, suggesting that PP242 inhibited cell proliferation and promoted cell survival in NB cells cultured under hypoxic conditions for 24 h (Table 3).

Taken together, our findings indicate that the mTOR inhibitor, PP242, can revert the gene expression changes induced by hypoxia in NB cells.

## 4. Discussion

About 50% of NB patients are classified as high-risk at diagnosis [2]. Despite multimodal treatment, the five-year overall survival for patients with high-risk NB is only 40% [2]. Approximately 20% of patients with high-risk NB progress early or are refractory to standard induction therapy, and 50% of patients who achieve remission later relapse [2]. Patients with relapsed and refractory NB have even poorer outcomes, with a five-year survival of less than 20% [2]. Therefore, new effective therapeutic drugs and therapeutic targets are needed to improve the survival of NB patients.

Hypoxia is a negative prognostic and predictive factor of treatment response due to its central role in tumor progression and resistance to therapy [26]. Accumulating evidence has demonstrated the central role of hypoxia in cancer metastasis, tumor cell transformation, de-differentiation, and immunomodulation in NB [5,27,28]. The role of the tumor microenvironment has acquired great attention for the generation of efficient therapeutic strategies to treat NB patients [29]. This increasing interest in targeting the tumor microenvironment has made tumor hypoxia one the most promising targets of therapy in several types of tumors [30], including NB [29]. Meanwhile, hypoxia-targeted treatment strategies have progressed into clinical trials [4], although many of these approaches have not been successfully implemented in the clinic, largely because trial designs have not included methods to identify and select patients with hypoxic tumors prior to enrolment. Therefore, the necessity to include reliable biomarkers that could be predictive of a positive response to hypoxia-targeted treatment strategies in future clinical trials has been pointed out [4]. We previously designed a biology-driven approach to assess the hypoxic status of NB primary tumors from the analysis of the gene expression profile of 11 distinct NB cell lines cultured under hypoxic and normoxic conditions for 18 h, deriving a robust 62 probesets signature (NB-hypo). NB-hypo was associated with lower survival in NB patients with hypoxic tumors with respect to those with non-hypoxic tumors [10]. Recently, we made a step toward the identification of a hypoxia biomarker in NB tumors by defining the NB-hop hypoxia signature, which allows for determining NB tumor hypoxia robustly using its gene expression profile [8]. Hypoxic tumors predicted by the NB-hop signature were positively associated with HIF-1α, but not with HIF-2α expression [8]. In the same work, we showed that hypoxia is an unfavorable prognostic factor for NB, as it induces profound metabolic changes in the tumor microenvironment and alters key cellular pathways and biological processes in NB tumors [8]. In addition, we have identified a set of genes whose expression is significantly altered in response to hypoxia in NB tumors. The subset of genes modulated by hypoxia and functionally involved in NB development and progression was used in the CMap analysis as the reference signature to find novel potential compounds counteracting the negative effect of hypoxia in NB.

The overarching assumption of our study is that compounds able to revert the expression of genes previously shown to be modulated by hypoxia in NB cells may revert the malignant phenotypes of hypoxic tumors. As a consequence, those compounds would represent new potential candidates for the treatment of NB patients requiring new effective therapeutic strategies. This type of assumption is at the basis of the design and functioning of the CMap technique [16], which is an effective and widely popular approach for drug repurposing [15].

We demonstrated that a data-driven analysis based on CMap represented a suitable approach for identifying new candidate compounds for hypoxia-targeted NB therapy from thousands of options. In fact, CMap analysis identified PI3K/Akt/mTOR inhibitors as potentially suitable candidate compounds for NB treatment, because they were able to revert the expression of genes in different cancer cell lines that we previously found to be associated with hypoxia in NB patients. The homogeneous negative connectivity score observed within the class of PI3K/Akt/mTOR inhibitors across the nine human cell lines suggests that these drugs have a potential efficacy in counteracting the negative effects of hypoxia, even though the effect may vary greatly among distinct compounds. This was particularly true for a subset of mTOR inhibitors, including KU-0063794, wortmannin, OSI-027, AZD-8055, WYE-125132, temsirolimus, and NVP-BEZ235.

As the cell lines used to create the CMap reference database did not include NB cells and have not been cultured under hypoxia conditions, we decided to validate the negative connectivity between mTOR inhibition and response to hypoxia using the gene expression profile of SK-N-BE(2)c NB cell line cultured under hypoxia for 24 h and treated with the mTOR inhibitor, PP242, or DMSO, as reported by Mohlin et al. [22]. The main reasons for us analyzing the gene expression profile of NB cell lines cultured under hypoxic conditions for 24 h was related to our interest in searching for compounds targeting acute/intermittent hypoxia in NB and because 24 h was the time point reported in the Mohlin dataset that most closely matched the time point we used to analyze the modulation of NB cell gene expression in response to acute hypoxia [8,10]. Our analyses showed a clear opposite expression of the genes differentially regulated by hypoxia in primary NB tumors in the SK-N-BE(2)c NB cell lines cultured under hypoxic conditions for 24 h followed by PP242 treatment, thereby validating the high opposite similarity observed with the CMap analysis.

We also observed the concordance of the gene expression between the genes up-regulated by hypoxia in primary NB tumors and those up-regulated by hypoxia in SK-N-BE(2)c cells cultured under hypoxia. An analysis based on GSEA used a custom gene set collection that was specifically designed to assess this concordance. GSEA was also employed with the Hallmark gene set collection to assess the extent to which the mTOR inhibitor, PP242, was able to revert hypoxia-induced changes in SK-N-BE(2)c NB cell lines. Analyses indicated that the PP242 compound reverted the enrichment of specific hallmark gene sets related to the cellular response to hypoxia, such as glycolysis and cell proliferation, but also the genes involved in mTORC1 signaling, which are compatible with mTOR inhibition and the suppression of cellular response to hypoxia [31].

Taken together, our findings point out a clear connectivity between PI3K/Akt/mTOR inhibitors and the cellular response to hypoxia, thereby representing a valid treatment perspective for NB.

The PI3K/Akt/mTOR signaling pathway is one of the major aberrantly activated intracellular pathways in human cancers, which plays an important role in the regulation of tumor cell proliferation, survival, response to stress, metabolism, motility, angiogenesis, and resistance to therapies [17,18]. The core components of this pathway, namely PI3Ks, AKT, and mTOR, have been extensively studied and found to be frequently hyperactivated in several human cancers, and their inhibition has been shown to lead to tumor regression in many preclinical studies [32,33]. Numerous selective pharmacological inhibitors of the PI3K/Akt/mTOR pathway have being developed, many of which are currently being tested in clinical trials, and are reported to be effective in the treatment of several types of cancer by exerting antiproliferative and proapoptotic effects and restoring tumor cell sensitivity to chemotherapy, radiotherapy, and hormonal treatment, thus showing great potential for targeted antitumor treatment [33,34,35].

Interestingly, several studies in the past years have implicated the PI3K/Akt/mTOR pathway in the regulation of cell responses to hypoxia, and the antitumor activity of PI3K/Akt/mTOR inhibitors appears to be mediated, at least in part, through the inhibition of cell responses to the hypoxic stress. Growing evidence, in fact, has demonstrated that HIF and PI3K/Akt/mTOR pathways act in an integrated way, influencing each other and the common downstream signaling pathways, increasing gene expression, cell metabolism and survival, tumorigenesis, and tumor growth [36]. PI3K/Akt/mTOR signaling was shown to regulate HIF-1α activation by hypoxia, as well as HIF1α-mediated resistance to hypoxia-induced apoptosis, in various human adult and pediatric cancers, including rhabdomyosarcoma and Ewing’s sarcomas [37], nasopharyngeal, colorectal, and glioma tumors [38]. The migration and invasion of human glioblastoma cells was enhanced under hypoxic conditions through the activation of the PI3K/Akt/mTOR pathway by targeting HIF-1 [39]. In addition, HIF-1α protein stabilization and the increased expression of its target gene, VEGF, was evident in uveal melanoma cells through a mTOR-dependent mechanism, and was shown to promote the invasive capacity of uveal melanoma cells through the AKT signaling pathway [40]. The effects of mTOR on HIF-1 stabilization and transactivating functions was also reported in prostate cancer cells cultured under hypoxic conditions [41]. Pharmacological targeting of the PI3K/mTOR pathway by specific inhibitors in human nasopharyngeal, colorectal, and glioma cells was effective in diminishing tumor hypoxia by reducing mitochondrial oxygen consumption [38]. These agents were also shown to significantly decrease hypoxia-induced HIF-1α protein expression, DNA binding, and transcription activity in human glioblastoma and prostate cancer cells, suppressing their migration and invasion [38,39], and to sensitize rhabdomyosarcoma and Ewing’s sarcomas to TRAIL- or doxorubicin-induced apoptosis under hypoxia [37]. These data, thus, provide a basis for the effective use of PI3K/Akt/mTOR pharmacological inhibitors for counteracting hypoxia effects on tumor cells.

Recent evidence demonstrates that the activation of the PI3K/Akt/mTOR pathway is also implicated in the pathogenesis of NB, and is correlated with tumor progression and a poor prognosis [42,43]. The potential efficacy of inhibiting the PI3K/Akt/mTOR pathway has been observed in NB preclinical studies [38,44,45,46], and several strategies have been developed to interfere with distinct components of this pathway, showing promises for molecular targeted therapies in advanced stage NB tumors [42,45,47]. However, the connection between PI3K/Akt/mTOR inhibitors and hypoxia remains poorly investigated in NB. It was shown that the mTOR complex 1 (mTORC1) upregulates the HIF-1 expression by promoting the glycolysis in the SK-N-BE(2)c cell line [22]. In addition, activation of the PI3K/Akt/mTOR pathway by growth factors, including insulin-like growth factor (IGF)-1, was reported to lead to mTOR phosphorylation, increased HIF-1α expression, and to induce HIF-1α-mediated VEGF transcription and secretion in distinct NB cell lines [48], which were blocked by mTOR or PI3K inhibition using specific small-molecule inhibitors [49], suggesting that targeting the PI3K/Akt/mTOR pathway has the potential to inhibit VEGF expression and limit NB tumor growth by inhibiting HIF-1 expression and activity. The data reported in this study add new important information, indicating that inhibitors of the PI3K/Akt/mTOR pathway can significantly revert the expression of the genes up- or down-regulated in NB hypoxic tumors, thus representing a potential target pathway to counteract the negative effects of hypoxia in NB.

Our analysis identified several potential candidate compounds for NB therapy, which include KU-0063794, wortmannin, OSI-027, AZD-8055, WYE-125132, temsirolimus, crizotinib, phorbol-12-myristate-13-acetate, triciribine, HG-5-113-01, BMS-754807, NVP-BEZ235, anisomycin, ivermectin, vincristine, and pyrvinium-pamoate. Temsirolimus, a well-known rapamicin rapalog, has been previously indicated as a novel adjuvant for the treatment of high-risk and relapsed NB patients [3,46,50], and has been successfully tested for NB in clinical trials, in combination with standard chemotherapy and monoclonal antibodies [51]. Vincristine and crizotinib are also currently used in the treatment of NB patients [46,52]. A combination of vincristine, topotecan, and doxorubicin was shown to improve the response rate of high-risk NB patients, as recently shown by our group [53]. However, the use of these compounds has never been conditioned to a previous assessment of the hypoxic status of the NB tumor. Therefore, the full potential of these compounds in NB has not been explored yet. The finding that a few compounds identified by our analysis have been previously proven to have therapeutic efficacy in NB confirm the reliability of this approach for the discovery of new drugs for NB treatment. Because temsirolimus, crizotinib, ivermectin, vincristine, ingenol, and pyrvinium-pamoate are FDA-approved drugs, they may be potentially used in new clinical trials. We believe that these compounds, in combination with other chemotherapeutic drugs or monoclonal antibodies, may improve the efficacy of treatments for NB patients, at both the time of diagnosis and/or relapse. Among the compounds with the highest connectivity with hypoxia identified in our CMap analysis, KU-0063794, wortmannin, OSI-027, AZD-8055, WYE-125132, phorbol-12-myristate-13-acetate, triciribine, HG-5-113-01, BMS-754807, NVP-BEZ235, anisomycin, ivermectin, ingenol, and pyrvinium-pamoate have never been used in studies involving NB patients or NB cell lines, thus representing new potential therapeutic drugs for NB.

mTOR is a serine/threonine kinase that acts through two structurally and functionally distinct protein complexes, mTORC1 and mTORC2, to sense and integrate multiple intracellular and environmental signals [54]. In their study, Mohlin and colleagues reported that PI3K-mTORC2, but not PI3K–mTORC1, regulates HIF-2α expression, suggesting a differential regulation of HIF-1α and HIF-2α by mTORC1 and mTORC2 complexes [22]. Several reports indicated that HIF-1α is dependent on both mTORC1 and mTORC2, whereas HIF-2α is dependent only on mTORC2 [55]. mTOR inhibitors KU-0063794, OSI-027, AZD-8055, WYE-125132, NVP-BEZ235, and wortmannin are potent dual mTORC1/mTORC2 inhibitors or provide dual inhibition on PI3K and mTOR signaling [56,57,58,59,60,61]. On the contrary, temsirolimus is the only one affecting mTORC1, but not mTORC2 [50,54]. mTOR inhibitors with the dual inhibition on PI3K and mTOR signaling, such as PP242, or mTOR kinase inhibitors, such as AZD-8055, were reported to be more effective and less prone to induce drug resistance than rapamicin rapalogs, such as temsirolimus [54].

Hypoxia tolerance is an important mechanism of adaptation used by cancer cells to survive under O_2_ reduced availability. Diverse cellular mechanisms that promote hypoxia tolerance have been reported, although they have only been partially characterized because of their complexity [36]. Unfolded protein response (UPR) is one of the strongest mechanisms activated by cancer cells to induce hypoxia tolerance [36,62]. We assessed the status of modulation of the genes involved in UPR by hypoxia by performing a new GSEA analysis. The analysis was carried out by comparing the gene expression profile of NB cells cultured under normoxic conditions with that of NB cells cultured under hypoxia, as reported by Mohlin and colleagues [22]. The enrichment results evidenced a significant up-regulation of the genes involved in UPR in NB cells cultured under hypoxia, indicating that hypoxia tolerance may be induced by hypoxia in NB cells. However, both mTORC1 signaling genes and the genes involved in UPR were significantly down-regulated in NB cells cultured under hypoxia and treated with the mTOR inhibitor, PP242, thereby suggesting that the inhibition of mTOR under hypoxia does not promote UPR-induced tolerance in NB cells.

Because PP242 was absent from the list of compounds in the CMap reference database, we could not show a direct connection between this specific mTOR inhibitor and hypoxia-modulated genes. Additional in vitro and in vivo experiments will be performed in the future to further investigate the efficacy of PI3K/Akt/mTOR inhibitors in hypoxic environments in NB.

## 5. Conclusions

PI3K/Akt/mTOR inhibitors represent a potential effective class of compounds targeting hypoxia in NB. PI3K/Akt/mTOR inhibitors may thus find future applicability as a new adjuvant therapy in randomized clinical trials involving NB patients with hypoxic tumors. The NB-hop signature may be instrumental to predict NB patients with an unfavorable prognosis who may benefit from treatment with PI3K/Akt/mTOR inhibitors in combination with other therapeutic strategies.

## Figures and Tables

**Table 1:** New candidate CMap compounds potentially targeting hypoxia-modulated genes in NB.

Id ^a^	Name ^b^	Description ^c^	Target ^d^	median_tau_score ^e^	FDA- Approved ^f^	Clinical Phase ^g^	Clinical Trial on NB ^h^
BRD-K67566344	KU-0063794	mTOR inhibitor	MTOR	−97.04	No	Preclinical	No
BRD-A75409952	wortmannin	PI3K inhibitor, ATM kinase inhibitor, ATR kinase inhibitor, DNA dependent protein kinase inhibitor, mTOR inhibitor, phosphatidylinositol 3-kinase (PI3K) inhibitor, and PLK inhibitor	PIK3CA, PIK3CG, PLK1, ATM, ATR, MTOR, PI4KA, PI4KB, PIK3CD, PIK3R1, PLK3, and PRKDC	−95.66	No	Preclinical	No
BRD-K94294671	OSI-027	mTOR inhibitor	MTOR	−94.93	No	1	No
BRD-K69932463	AZD-8055	mTOR inhibitor	MTOR	−94.49	No	1	No
BRD-A45498368	WYE-125132	mTOR inhibitor and PI3K inhibitor	MTOR and PIK3CA	−94.32	No	Preclinical	No
BRD-A62025033	temsirolimus	mTOR inhibitor, cell cycle inhibitor, immunosuppressant, and protein kinase inhibitor	MTOR and PTEN	−93.9	Yes	Launched	Yes
BRD-K78431006	crizotinib	ALK tyrosine kinase receptor inhibitor, c-Met inhibitor, hepatocyte growth factor receptor inhibitor, and tyrosine kinase inhibitor	ALK, MET, CYP2B6, CYP3A5, MST1R, and ROS1	−93.21	Yes	Launched	Yes
BRD-A15079084	phorbol-12-myristate-13-acetate	PKC activator and CD antagonist	CD4, KCNT2, PRKCA, and TRPV4	−93.05	No	2	No
BRD-A42649439	triciribine	AKT inhibitor	AKT1, AKT2, and AKT3	−93.04	No	1 and 2	No
BRD-U82589721	HG-5-113-01	Protein kinase inhibitor	ABL1, LTK, and STK10	−92.49	No	N.D.	No
BRD-K13049116	BMS-754807	Insulin growth factor receptor inhibitor	IGF1R and AKT1	−92.34	No	2	No
BRD-K12184916	NVP-BEZ235	mTOR inhibitor, PI3K inhibitor, and protein kinase inhibitor	MTOR, PIK3CA, PIK3CG, PIK3CD, ATR, and PIK3CB	−91.92	No	3	No
BRD-K91370081	anisomycin	DNA synthesis inhibitor	NHP2L1, RPL10L, RPL11, RPL13A, RPL15, RPL19, RPL23, RPL23A, RPL26L1, RPL3, RPL37, RPL8, and RSL24D1	−91.61	No	Preclinical	No
BRD-A48570745	ivermectin	GABA receptor agonist, acetylcholine receptor allosteric modulator, GABA release stimulant, and purinergic receptor allosteric modulator	CHRNA7, GABRB3, GLRA3, and P2RX7	−91.41	Yes	Launched	No
BRD-A60414806	vincristine	Tubulin inhibitor, microtubule inhibitor, microtubule polymerization inhibitor, tubulin polymerisation inhibitor, and vinca alkaloid	TUBB and TUBA4A	−90.83	Yes	Launched	Yes
BRD-A11678676	wortmannin	PI3K inhibitor, ATM kinase inhibitor, ATR kinase inhibitor, DNA dependent protein kinase inhibitor, mTOR inhibitor, phosphatidylinositol 3-kinase (PI3K) inhibitor, and PLK inhibitor	PIK3CA, PIK3CG, PLK1, ATM, ATR, MTOR, PI4KA, PI4KB, PIK3CD, PIK3R1, PLK3, and PRKDC	−90.73	No	Preclinical	No
BRD-A52650764	ingenol	PKC activator	PRKCD and PRKCE	−90.37	Yes	Launched	No
BRD-M86331534	pyrvinium-pamoate	AKT inhibitor, androgen receptor (AR) inhibitor, and androgen receptor antagonist	AR	−90.28	Yes	Launched	No
BRD-K87343924	wortmannin	PI3K inhibitor, ATM kinase inhibitor, ATR kinase inhibitor, DNA dependent protein kinase inhibitor, mTOR inhibitor, phosphatidylinositol 3-kinase (PI3K) inhibitor, and PLK inhibitor	PIK3CA, PIK3CG, PLK1, ATM, ATR, MTOR, PI4KA, PI4KB, PIK3D, PIK3R, PLK3, and PRKDC	−90.06	No	Preclinical	No

^a^ Id indicates the Broad Institute compound identifier. ^b^ Name indicates the vendor compound name. ^c^ Description indicates the main mechanisms of action of a compound. ^d^ Target lists the reported target genes of a drug. ^e^ Median_tau_score summarizes the connectivity between a Query signature and a drug across multiple cell types. The drugs in the table are sorted by increasing order of median tau score. ^f^ FDA-approved indicates if a drug has been approved for commercialization. ^g^ Clinical phase indicates the trial phase of the drug, if any. We considered any disease. ^h^ Clinical trial on NB indicates the trial phase of the drug specifically focusing on NB. N.D. indicates not determined according to the [24] website or the Repurposing tool.

**Table 2:** Hallmark gene sets enriched in NB cell lines cultured under hypoxia for 24 h.

Gene Set ^a^	ES ^b^	NES ^c^	NOM *p*-Value ^d^	FDR q-Value ^e^
HALLMARK_HYPOXIA	0.74	2.96	0.00	0.00
HALLMARK_GLYCOLYSIS	0.65	2.64	0.00	0.00
HALLMARK_MTORC1_SIGNALING	0.65	2.60	0.00	0.00
HALLMARK_ANGIOGENESIS	0.65	1.92	0.00	0.00
HALLMARK_PROTEIN_SECRETION	0.47	1.73	0.00	0.01
HALLMARK_CHOLESTEROL_HOMEOSTASIS	0.51	1.71	0.00	0.01
HALLMARK_FATTY_ACID_METABOLISM	0.44	1.70	0.00	0.01
HALLMARK_UNFOLDED_PROTEIN_RESPONSE	0.45	1.64	0.00	0.01
HALLMARK_ADIPOGENESIS	0.40	1.56	0.00	0.02
HALLMARK_G2M_CHECKPOINT	−0.43	−1.62	0.00	0.02
HALLMARK_MITOTIC_SPINDLE	−0.58	−2.19	0.00	0.00

^a^ Column displays significantly enriched gene sets belonging to the MSigDB Hallmark collection. Enrichment analysis was performed with the GSEA tool using the gene expression profile of NB cell lines cultured in normoxic or hypoxic conditions for 24 h. Data have been unlogged prior to any analysis. ^b^ ES (enrichment score) indicates the maximum deviation from zero encountered in a random walk for a gene set. ^c^ NES (normalized enrichment score) indicates the fraction between the ES and the mean of the ES against 1000 permutations of the gene sets. Gene sets are sorted by decreasing order of NES. ^d^ NOM *p*-value indicates the probability that an ES equal to or higher than that found for the gene set may be observed by chance. Gene sets with a NOM *p*-value lower than 0.05 are considered significant. ^e^ FDR q-value is the estimated probability that the normalized enrichment score represents a false positive finding. Values ≤ 0.05 are considered acceptable.

**Table 3:** Hallmark gene sets enriched in NB cell lines treated for 24h with the mTORC inhibitor.

Gene Set ^a^	ES ^b^	NES ^c^	NOM *p*-Value ^d^	FDR q-Value ^e^
HALLMARK_E2F_TARGETS	−0.87	−2.88	0.00	0.00
HALLMARK_G2M_CHECKPOINT	−0.80	−2.62	0.00	0.00
HALLMARK_MTORC1_SIGNALING	−0.75	−2.50	0.00	0.00
HALLMARK_GLYCOLYSIS	−0.61	−2.07	0.00	0.00
HALLMARK_MYC_TARGETS_V1	−0.61	−2.05	0.00	0.00
HALLMARK_ESTROGEN_RESPONSE_LATE	−0.59	−1.98	0.00	0.00
HALLMARK_CHOLESTEROL_HOMEOSTASIS	−0.66	−1.95	0.00	0.00
HALLMARK_UV_RESPONSE_UP	−0.58	−1.91	0.00	0.00
HALLMARK_REACTIVE_OXYGEN_SPECIES_PATHWAY	−0.69	−1.87	0.00	0.00
HALLMARK_MITOTIC_SPINDLE	−0.54	−1.82	0.00	0.00
HALLMARK_PI3K_AKT_MTOR_SIGNALING	−0.55	−1.76	0.00	0.00
HALLMARK_SPERMATOGENESIS	−0.55	−1.74	0.00	0.00
HALLMARK_UNFOLDED_PROTEIN_RESPONSE	−0.54	−1.71	0.00	0.00
HALLMARK_OXIDATIVE_PHOSPHORYLATION	−0.51	−1.68	0.00	0.00
HALLMARK_DNA_REPAIR	−0.52	−1.66	0.00	0.00
HALLMARK_FATTY_ACID_METABOLISM	−0.49	−1.61	0.00	0.00
HALLMARK_ANDROGEN_RESPONSE	−0.51	−1.57	0.00	0.01
HALLMARK_MYC_TARGETS_V2	−0.54	−1.54	0.01	0.01
HALLMARK_HYPOXIA	−0.46	−1.53	0.00	0.01
HALLMARK_XENOBIOTIC_METABOLISM	−0.45	−1.49	0.00	0.01
HALLMARK_APOPTOSIS	−0.44	−1.46	0.01	0.02
HALLMARK_ADIPOGENESIS	−0.43	−1.44	0.00	0.02
HALLMARK_PEROXISOME	−0.46	−1.41	0.02	0.03

^a^ Column displays significantly enriched gene sets belonging to the MSigDB Hallmark collection. An enrichment analysis was performed with the GSEA tool using the gene expression profile of NB cell lines cultured in hypoxic conditions for 24 h and then treated with DMSO or the mTORC complex inhibitor PP242. Data have been unlogged prior to any analysis. ^b^ ES (enrichment score) indicates the maximum deviation from zero encountered in a random walk for a gene set. ^c^ NES (normalized enrichment score) indicates the fraction between the ES and the mean of the ES against 1000 permutations of the gene sets. Gene sets are sorted by increasing order of NES. ^d^ NOM *p*-value indicates the probability that an ES equal to or higher than that found for the gene set may be observed by chance. Gene sets with a NOM *p*-value lower than 0.05 are considered significant. ^e^ FDR q-value is the estimated probability that the normalized enrichment score represents a false positive finding. Values ≤ 0.05 are considered acceptable.

## Data Availability

CMap data used in the present are publicly accessible in the CMap repository [19]. The gene expression profile of the NB cell lines analyzed in this study are publicly accessible in R2: Genomics Analysis and Visualization Platform [23], and in the Gene Expression Omnibus (GEO) repository using accession number GSE69833.

## Supplementary Materials

The following are available online at https://www.mdpi.com/article/10.3390/cancers13112809/s1, Table S1: Genes whose modulation was significant in the subset of UF NB-hop patients. Table S2: Connectivity results between hypoxia-modulated genes in NB tumors and compounds in the CMap reference database. Table S3: Presence/absence of hypoxia-modulated genes in the Mohlin dataset.
